# The effect of intermittent versus continuous enteral feeding for critically ill patients: a meta-analysis of randomized controlled trials

**DOI:** 10.3389/fnut.2023.1214774

**Published:** 2023-08-21

**Authors:** Jing Qu, Xiaoya Xu, Chaobo Xu, Xuzhong Ding, Kai Zhang, Leshuang Hu

**Affiliations:** ^1^Department of General Surgery, Lishui People’s Hospital, Lishui, China; ^2^Department of Critical Care Medicine, Second Affiliated Hospital, Zhejiang University School of Medicine, Hangzhou, China

**Keywords:** intermittent feeding strategy, continuous feeding strategy, enteral nutrition, critically ill, meta - analysis

## Abstract

**Objectives:**

The appropriate strategy for enteral feeding in critically ill patients still remains controversial. Therefore, we conducted this meta-analysis to compare the effect of intermittent versus continuous enteral feeding method for critically ill patients.

**Methods:**

Electronic databases including PubMed, Embase, Scopus, and Cochrane Library were searched up to April 10th, 2023 for randomized controlled trials evaluating the effect of intermittent versus continuous enteral feeding for critically ill patients. The primary outcomes were feeding intolerances, including diarrhea, vomiting, distension, constipation, gastric retention, and aspiration pneumonia. The secondary outcomes were mortality in intensive care unit (ICU), length of stay in ICU, and achievement of nutritional goal.

**Results:**

Thirteen studies with a total of 884 patients were analyzed in this meta-analysis. Overall, the use of intermittent enteral feeding was associated with higher incidence of diarrhea (OR 1.66, 95%CI 1.13 to 2.43, I^2^ = 16%) and distension (OR 2.29, 95%CI 1.16 to 4.51, I^2^ = 0%), lower incidence of constipation (OR 0.58, 95%CI 0.37 to 0.90, I^2^ = 0%), and longer length of ICU stay (MD 1.09, 95%CI 0.53 to 1.64, I^2^ = 0%). Moreover, no significant difference was identified for other outcome measures.

**Conclusion:**

In critically ill patients, the implementation of intermittent enteral feeding was associated with higher incidence of diarrhea and distension, longer length of ICU stay, but lower occurrence of constipation. Nevertheless, the absence of sufficient high-quality randomized controlled clinical trials precludes any definitive conclusions regarding the optimal approach to enteral feeding in this population. There is an imperative need for more studies to further assess the efficacy of the two enteral feeding strategies.

## Introduction

Nutritional support is important for critically ill patients in intensive care unit (ICU), adequate nutritional therapy can improve clinical outcomes associated with malnutrition (1, 2). The methods of nutritional support have been widely described, including enteral nutrition (EN) and parenteral nutrition (PN) (3). EN is safer and more cost-effective than PN, and EN has the potential advantage of maintaining gastrointestinal tract integrity to prevent bacterial translocation (4, 5). Thus, current clinical guidelines promote the early implementation of EN for critically ill patients (2). However, critically ill patients are commonly affected by gastrointestinal dysfunction due to various factors such as post-operative ileus, gastric stasis, gut hypoperfusion, and administration of certain antibiotics and sedatives (6). These factors lead to an increased occurrence of feeding intolerance, which in turn hinders the successful implementation of enteral nutrition. Thus, there is an urgent need for further research to establish the optimal method of delivering EN and to address the discrepancies between guidelines and clinical practices (7).

There are two major methods for EN administration: continuous and intermittent enteral feeding. Continuous feeding uses a constant rate hourly by an electric feeding pump for 24 h per day (8). The infusion speed is relatively low and is therefore theorized as a safer enteral nutrition delivery strategy particularly for patients with an intolerance of enteral feeding (9). However, continuous enteral feeding was associated with a reduction in patient mobility and alterations in gastrointestinal hormone secretion, which may further lead to long-term metabolic complications such as hyperglycemia and insulin resistance (10, 11). Conversely, intermittent feeding delivers nutrition multiple times per day (generally four to six times a day) by a feeding pump, syringe, or gravity pump (8). It has been considered as a more physiological approach compared to continuous strategy, as it provides patients with greater mobility, increases protein synthesis, helps maintain regular secretion and digestion of gastrointestinal hormones (11, 12). Nevertheless, current clinical guideline and studies indicated that intermittent feeding increased the risk of feeding intolerance for critically ill patients (2, 6, 13, 14). A recent randomized trial indicated that compared with intermittent feeding, continuous feeding significantly improved the achievement of target nutrition requirements (15). The effectiveness and safety of the two enteral feeding methods for in critically ill patients still remain controversial, it is imperative to determine the optimal enteral nutrition strategy. Therefore, we aimed to perform a meta-analysis of randomized controlled trials (RCTs) to compare the effect of intermittent versus continuous enteral feeding in critically ill patients.

## Methods

### Study selection

This meta-analysis was performed according to the updated PRISRMA statement (16), the PRISRMA checklist is shown in Supplementary material 1. We preregistered our study protocol in Open Science Framework.^1^ Two authors (JQ, XX) searched the PubMed, Embase, Scopus, and Cochrane Library for relevant studies in English up to April 10th, 2023. The search algorithms included “intermittent,” “continuous,” “critically ill,” and “randomized.” The details of the search strategies are presented in Supplementary material 2.

### Inclusion criteria

Studies fulfilled the inclusion criteria were included:

1. Type of study: randomized trials;
2. Population: critically ill patients required enteral nutritional support;
3. Intervention: intermittent enteral feeding;
4. Comparison: continuous enteral feeding;
5. Outcomes: trial must have reported at least one outcome of interest as detailed below.

### Outcomes

The primary outcomes were feeding intolerances, including diarrhea, vomiting, distension, constipation, gastric retention, and aspiration pneumonia. The secondary outcomes were mortality in ICU, length of stay in ICU, and achievement of nutritional goal. The definitions of outcomes were the same as in the included trials.

### Data extraction and quality assessment

Two authors (JQ, LH) separately screened all retrieved studies, then extracted the relevant information (first author or study name, publication years, study design, population, intervention and control methods). Each clinical outcome of this meta-analysis was also extracted from each included study.

Two authors (JQ, LH) adopted the Cochrane risk of bias tool (17) to assess the methodological quality of including studies. Any disagreement between the two authors was resolved by a consensus after discussing with a third adjudicator (XX).

### Statistical synthesis and analysis

The computation of pooled odds ratios (OR) with corresponding 95% confidence intervals (CI) for dichotomous outcomes, and mean difference (MD) with 95% CI for continuous outcomes. The heterogeneity was assessed by the Higgins inconsistency (I^2^) statistics (18). Substantial heterogeneity was identified when I^2^ value>30% and a random-effects model was employed to perform the analysis, otherwise a fixed-effects model would be used. In addition, publication bias was evaluated through the use of the funnel plot and Egger’s regression test (19). Furthermore, a sensitivity analysis was performed through the consecutive exclusion of each study to investigate the effect of individual studies. All statistical analyses and assessments of bias risk were conducted by Review Manager Version 5.3, “meta” package in R software (version 4.3.1).

## Results

### Study characteristics

**Figure 1:**
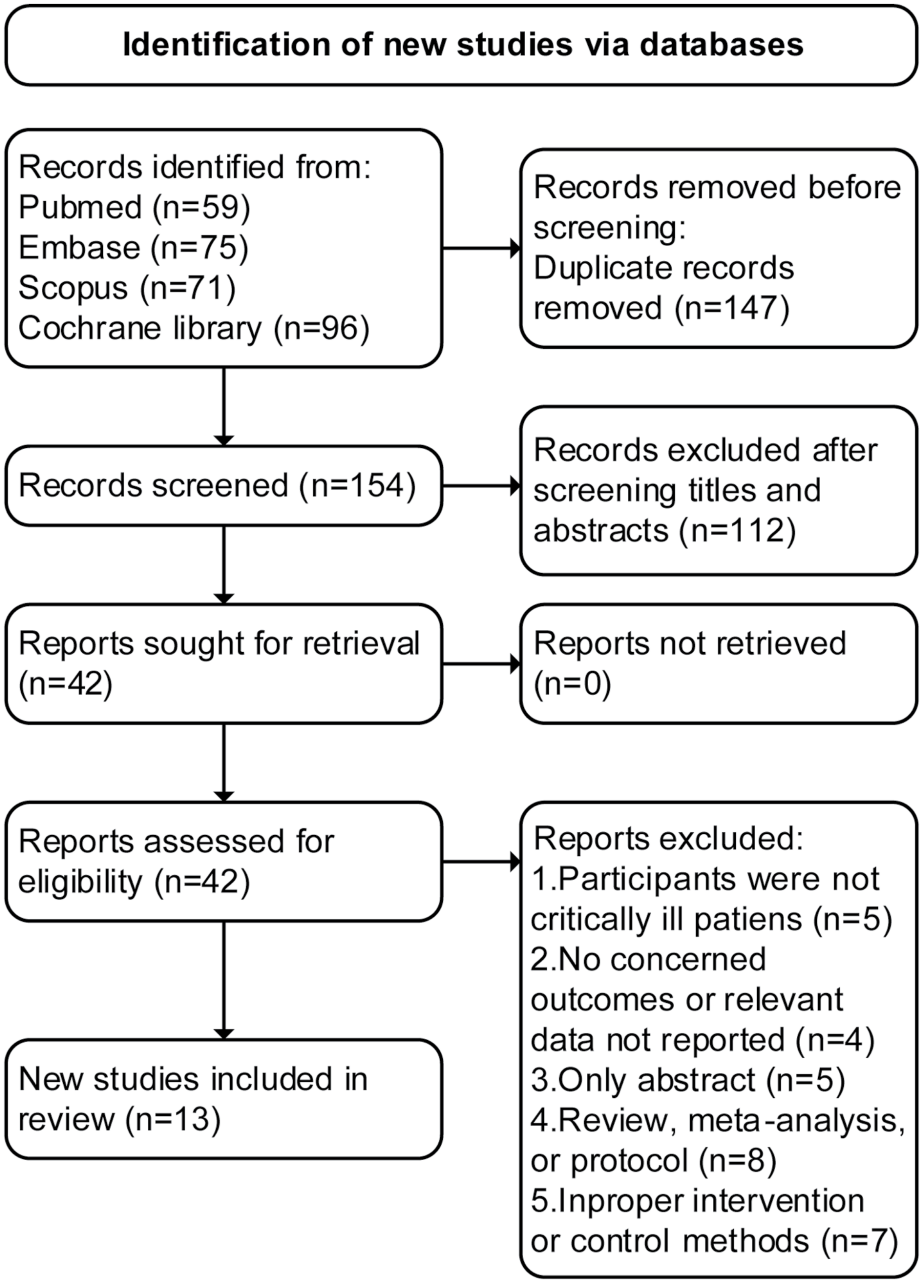
PRISMA 2020 flow diagram for the meta-analysis.

An initial search of the literature resulted in the identification of 301 articles, of which 147 were deemed as duplicates and excluded. Through the screening of abstracts, an additional 112 studies were excluded. Following a thorough evaluation of the full text, 29 additional studies were excluded for various reasons. Finally, 13 RCTs (13, 15, 20–30) met the inclusion criteria and were included in this study (see Figure 1 for detailed follow chart).

The characteristics of the included studies are presented in Table 1. A total of 884 patients were analyzed, with 444 patients receiving intermittent enteral feeding and 440 patients receiving continuous enteral feeding during the respective study periods. The study periods ranged from 1 to 7 days. The number of patients in each study ranged from a minimum of 18 up to 160, and sample size of all included studies were relatively small (<100 patients per arm) (31). Patients were typically receiving enteral nutrition support in a mixed ICU. Seven studies reported the illness severity scores, with the average APACHE II score ranging from 12 to 28. The study durations ranged from 1 to 14 days, most of included studies compared the two feeding regimens in a 7-day study period.

**Table 1:** Characteristics of studies included in the meta-analysis.

Study and year	Design	Number of patients (Intermittent/Continuous)	Population	Characteristics (Intermittent/Continuous)	Study arm		Study period	Clinical outcomes
					Intermittent	Continuous		
Lee et al. (15)	Unblinded, single-center	49/50	Patients ≥20 years of age, required mechanical ventilation	Age: 66.2/67.5; Male (%): 67.3/66.0; BMI: 22.0/23.3; APACHE II: 27.7/28.6	1 h for 4 times/day, initial rate was 150 ml/h	24 h/day, initial rate was 25 ml/h	7 days	Feeding intolerances, achievement of nutritional goal, ICU mortality, length of ICU stay
Ren et al. (27)	Single-blinded, single-center	32/30	Patients admitted to ICU and fed through gastric tubes	Age: 66/55; Male (%): 53/63; BMI: 24/23; APACHE II: 19/16	2 h for 3 times/day	24 h/day	7 days	Feeding intolerances, ICU mortality, length of ICU stay
Zhu et al. (13)	Single-blinded, single-center	40/38	Patients ≥18 years of age, diagnosed with hemorrhagic stroke, GCS ≤12	Age: 59.9/59.6; Male: 55.3/47.5; BMI: 24.6/24.1	0.5–1 h for 4 times/day	24 h/day, maximum rate was 100 ml/h	7 days	Feeding intolerances
McNelly et al. (25)	Single-blinded, multicenter	62/59	Patients ≥18 years of age, required mechanical ventilation for ≥48 h	Age: 55.2/60.3; Male: 66.1/67.8; APACHE II: 23.1/20.2	Six bolus feeds, one bolus every 4 h	Total volume of feed administered over 24 h	10 days	Feeding intolerances, achievement of nutritional goal requirement, ICU mortality
Nasiri et al. (26)	Triple-blinded, single-center	20/20	Patients aged between 18 to 65 years, admitted to the ICUs with the diagnosis of sepsis	Age: 54.6/53.0; Male: 38.2/35.3	18 h/day, with night rest for 6 h	24 h/day	3 days	Feeding intolerances
Kadamani et al. (22)	Unblinded, single-center	15/15	Patients aged between 20 and 80 years, received mechanical ventilation and EN for ≥72 h	Age: 61.6/64.7; Male: 66.7/60.0; BMI: 23.3/23.1; APACHE II: 16.0/20.3	10–15 min every 4–6 h	24 h/day	3 days	Feeding intolerances
Tavares de Araujo et al. (30)	Unblinded, single-center	18/23	Patients over 18 years of age, under clinical treatment in an intensive care unit	Age: 68.9/61.3; Male: 55.6/60.9; BMI: 24.6/22.3; APACHE II: 20.7/22.4	18 h/day, with night rest for 6 h	24 h/day	5 days	Feeding intolerances, achievement of nutritional goal, ICU mortality
Maurya et al. (24)	Unblinded, single-center	20/20	Adult male patients age of 20–60 years with history of head injury requiring mechanical ventilatory support	Age: 40.2/40.7; Male: 100/100; BMI: 22.0/20.6	3 times/day	24 h/day	1 day	Feeding intolerances
MacLeod et al. (23)	Unblinded, single-center	79/81	Patients over 18 years of age, admitted to the trauma intensive care unit, required mechanical ventilation ≥48 h	Age: 44.6/48.4; Male: 67.1/74.1; APACHE II: 12/14	100 ml of formula within 30–60 min every 4 h	24 h/day, initial rate was 20 ml/h	7 days	Feeding intolerances, achievement of nutritional goal, ICU mortality, length of ICU stay
Chen et al. (21)	Unblinded, single-center	56/51	Patients over 20 years of age, admitted to the trauma intensive care unit, required mechanical ventilation	Male: 76.8/76.5	125 ml of formula every 4 h	24 h/day, initial rate was 25 ml/h	7 days	Feeding intolerances
Serpa et al. (28)	Unblinded, single-center	14/14	Critically ill patients admitted to ICU because of clinical or surgical emergencies	Age: 64.9/69.6; Male: 50.0/64.3	1 h for 6 times/day	24 h/day	3 days	Feeding intolerances, ICU mortality, length of ICU stay
Steevens et al. (29)	Unblinded, single-center	9/9	Multiple trauma patients age of 18–70 years admitted to ICU, expected to need enteral nutrition ≥5 days	Age: 35.9/37.3; Male: 55.6/77.8; BMI: 27.5/25.4	125 ml within 15 min every 4 h	24 h/day, initial rate was 25 ml/h	7 days	Feeding intolerances, achievement of nutritional goal
Bonten et al. (20)	Unblinded, single-center	30/30	All mechanically ventilated patients over 15 years of age, admitted to ICU	Age: 68/65; Male: 53.3/63.3; APACHE II: 17/19	18 h/day, with night rest for 6 h	24 h/day	14 days	Feeding intolerances, ICU mortality

BMI, body mass index; APACHE II, acute physiology and chronic health evaluation II; ICU, intensive care unit; GCS, Glasgow coma scale; EN, enteral nutrition.

### Quality assessment

**Figure 2:**
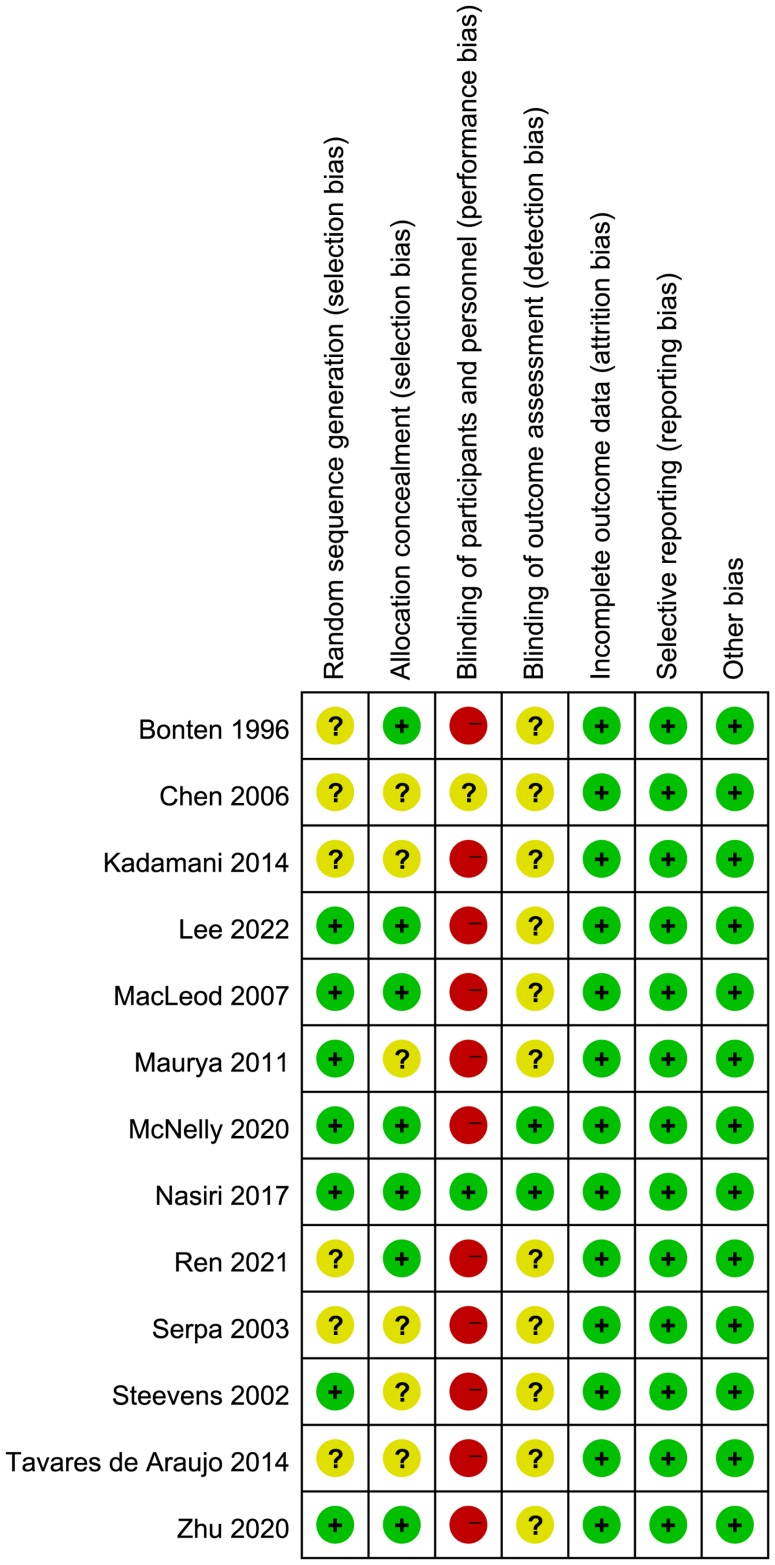
Assessment of quality by the Cochrane risk of bias tool.

The results of quality assessment (Figure 2) showed that most included trials were rated as high risk of bias, largely due to a lack of blinding and allocation concealment. Furthermore, 12 trials did not report the blinding method for outcome assessment, which may result in an underestimation or overestimation of the true effect.

In addition, we conducted an assessment of publication bias utilizing the Egger’s test and funnel plot, and the results did not indicate a significant risk of publication bias (Egger’s test, *p* > 0.05; Supplementary material 3).

### Primary outcomes

**Figure 3:**
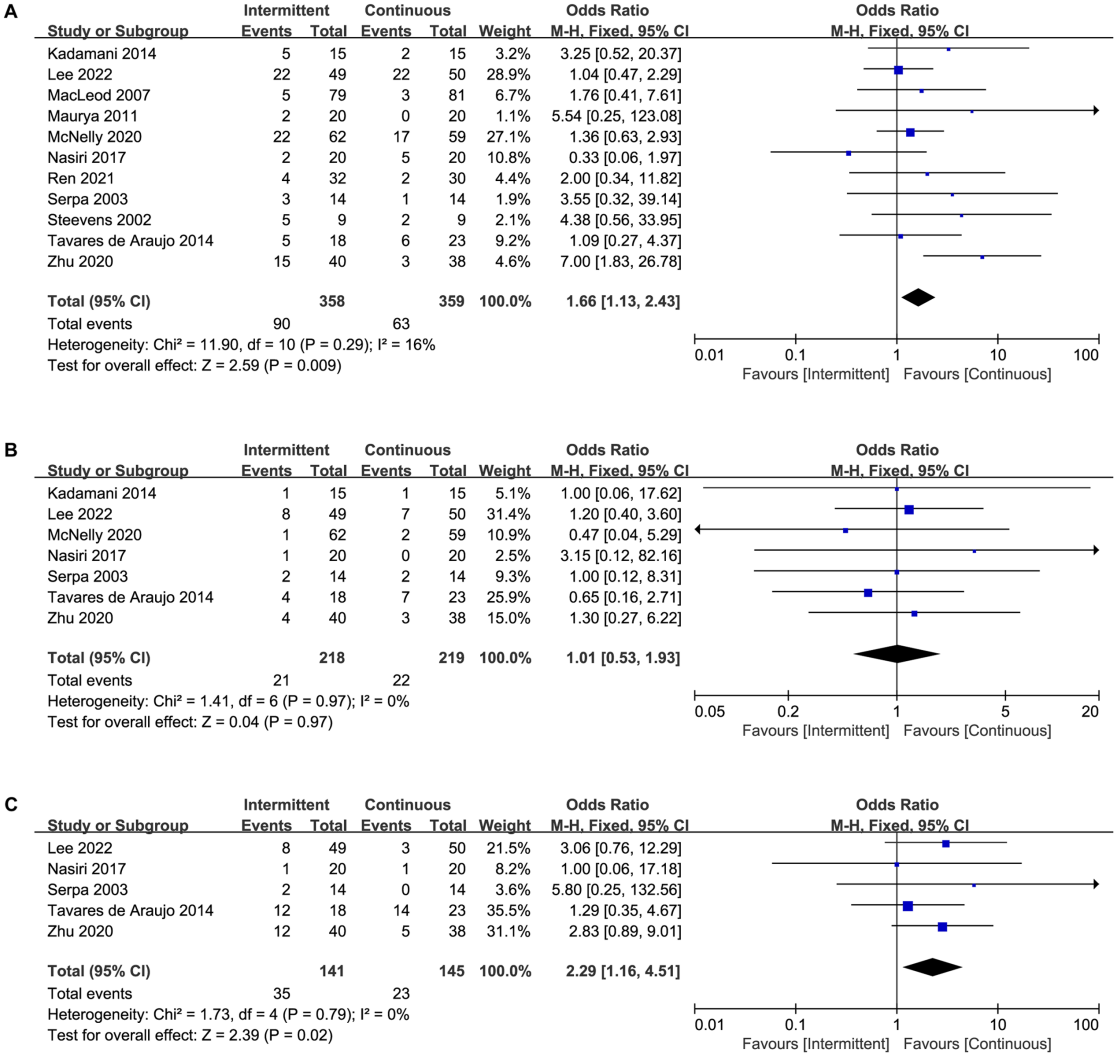
Forest plot showing the difference between intermittent versus continuous enteral feeding for **(A)** diarrhea, **(B)** vomiting, and **(C)** distension.

**Figure 4:**
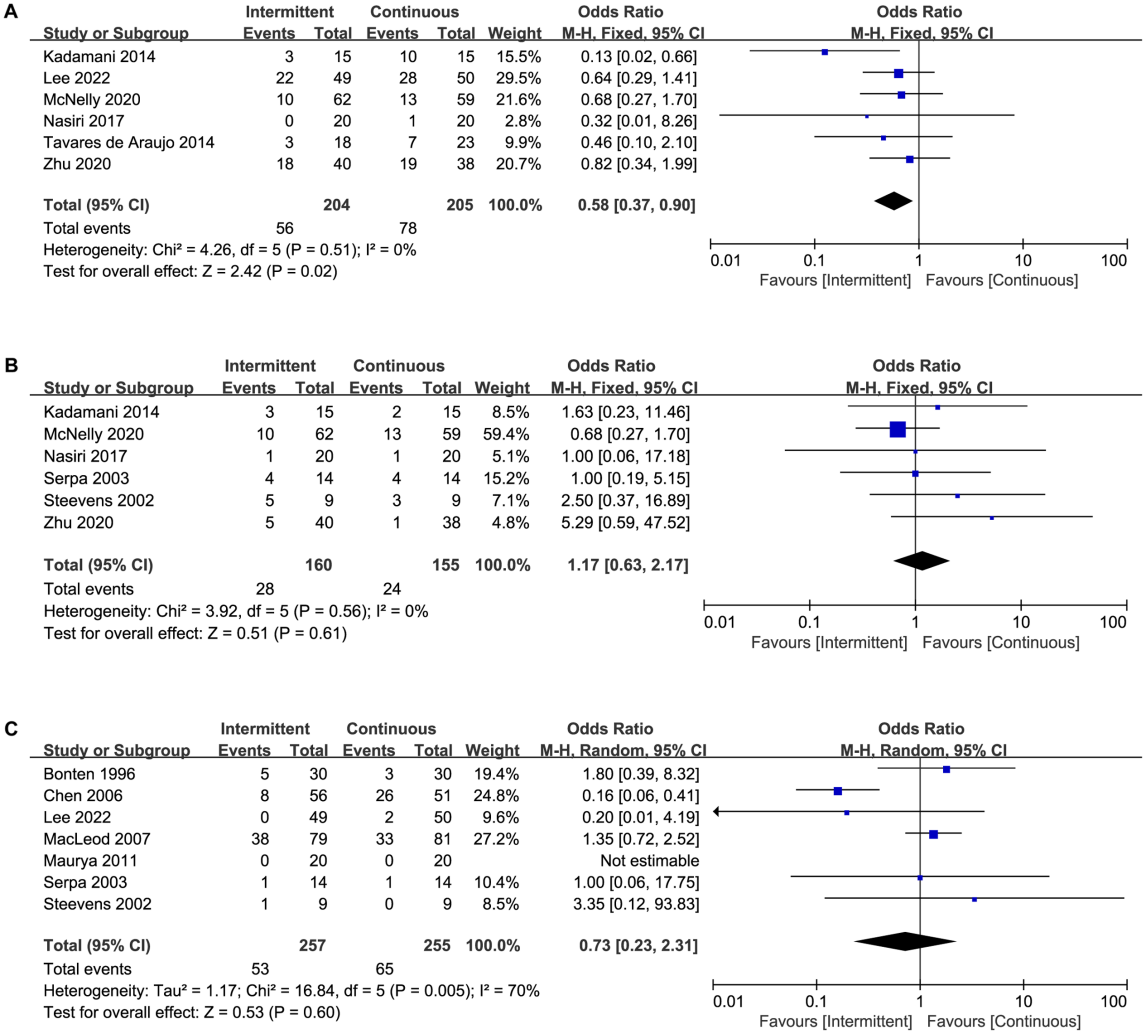
Forest plot showing the difference between intermittent versus continuous enteral feeding for **(A)** constipation, **(B)** gastric retention, and **(C)** aspiration pneumonia.

A total of 12 trials reported the incidence of feeding intolerances (Supplementary material 4), including 11 trials reported diarrhea (Figure 3A), seven trials reported vomiting (Figure 3B), five trials reported distension (Figure 3C), six trials reported constipation (Figure 4A), six trials reported gastric retention (Figure 4B), and seven trials reported aspiration pneumonia (Figure 4C). Overall, the pooled data showed that the use of intermittent enteral feeding was associated with higher incidence of diarrhea (OR 1.66, 95%CI 1.13 to 2.43, I^2^ = 16%, Figure 3A) and distension (OR 2.29, 95%CI 1.16 to 4.51, I^2^ = 0%, Figure 3C), lower incidence of constipation (OR 0.58, 95%CI 0.37 to 0.90, I^2^ = 0%, Figure 4A). No statistically significant difference was identified between intermittent and continuous enteral feeding for vomiting (OR 1.01, 95%CI 0.53 to 1.93, I^2^ = 0%, Figure 3B), gastric retention (OR 1.17, 95%CI 0.63 to 2.17, I^2^ = 0%, Figure 4B), and aspiration pneumonia (OR 0.73, 95%CI 0.23 to 2.31, I^2^ = 70%, Figure 4C).

However, when we assessed the effect of every single trial on the pooled result by leave-one-out sensitivity analysis, we found that exclusion of several studies might change the results. The incidence of diarrhea became no statistical significance when we excluded the trial by Zhu et al. (13) (Supplementary material 3). Similarly, excluding the trial by Lee et al. (15), Zhu et al. (13), or Kadamani et al. (20) changed the results of distension and constipation (Supplementary material 3). The sensitivity analysis showed that the results were not robust enough. Furthermore, no trial was found to have an undue influence on other outcomes as the effect size remained statistically significant on the exclusion of any trial (Table 2).

**Table 2:** Main findings of our meta-analysis.

Outcome	*N*	Result
Primary outcomes		
Diarrhea	11	OR 1.66, 95%CI 1.13 to 2.43, I^2^ = 16%
Vomiting	7	OR 1.01, 95%CI 0.53 to 1.93, I^2^ = 0%
Distension	5	OR 2.29, 95%CI 1.16 to 4.51, I^2^ = 0%
Constipation	6	OR 0.58, 95%CI 0.37 to 0.90, I^2^ = 0%
Gastric retention Low	6	OR 1.17, 95%CI 0.63 to 2.17, I^2^ = 0%
Aspiration pneumonia	7	OR 0.73, 95%CI 0.23 to 2.31, I^2^ = 70%
Secondary outcomes		
ICU mortality	7	OR 1.49, 95%CI 0.97 to 2.27, I^2^ = 0%
Length of ICU stay	4	MD 1.09, 95%CI 0.53 to 1.64, I^2^ = 0%
Achievement of nutritional goal	5	OR 1.99, 95%CI 0.67 to 1.45, I^2^ = 22%

OR, odds ratio; MD, mean difference; CI, confidence interval; ICU, intensive care unit.

### Secondary outcomes

**Figure 5:**
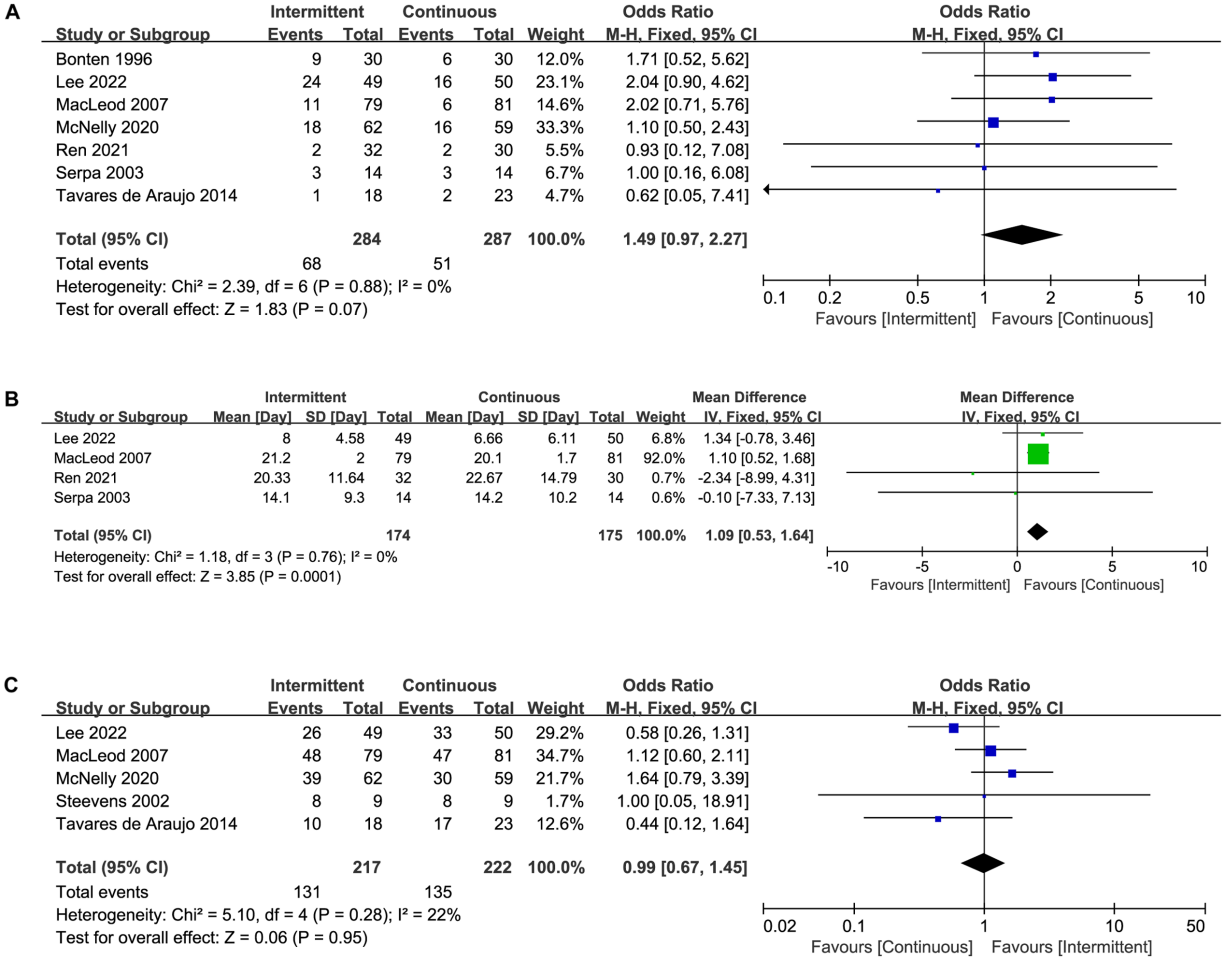
Forest plot showing the difference between intermittent versus continuous enteral feeding for **(A)** mortality in ICU, **(B)** length of ICU stay, **(C)** achievement of nutritional goal.

Mortality in ICU was reported in seven trials (Figure 5A), length of ICU stay was reported in four trials (Figure 5B), and achievement of nutritional goal was assessed in five trials (Figure 5C). There was a statistically significant difference between intermittent and continuous enteral feeding, with a prolonged length of ICU stay in patients receiving intermittent enteral feeding (MD 1.09, 95%CI 0.53 to 1.64, I^2^ = 0%, Figure 5B). No statistically significant difference was observed for mortality in ICU (OR 1.49, 95%CI 0.97 to 2.27, I^2^ = 0%, Figure 5A) or achievement of nutritional goal (OR 1.99, 95%CI 0.67 to 1.45, I^2^ = 22%, Figure 5C). Nevertheless, the difference in length of ICU became no statistically significant if we excluded the trial by MacLeod et al. (23), indicated the result was not robust enough.

## Discussion

Nutritional support is one of the important parts of treatments for critically ill patients (32). Despite its importance, there is limited evidence comparing the intermittent and continuous enteral feeding methods in clinical practice. In this study, we conducted a comprehensive review of existing literature that compares the effect of intermittent and continuous enteral feeding on feeding intolerance and clinical outcomes in critically ill patients. The findings of our meta-analysis indicated that intermittent enteral feeding was associated with increased risk of diarrhea and distension, as well as a longer ICU stay. Furthermore, the ICU mortality was higher in patients receiving intermittent feeding than others receiving continuous feeding, but the difference did not reach statistical significance. Conversely, a lower incidence of constipation was observed in the intermittent feeding group. However, there were no other differences in the incidence of vomiting, gastric retention, and aspiration pneumonia. Critically ill patients have high risk of developing feeding intolerance. Studies have shown that among critically ill patients, the rate of feeding intolerance can exceed 30 percent (33). In current clinical practice, both intermittent and continuous enteral feeding are commonly utilized and widely accepted as effective methods for delivering prescribed diets (9). However, current evidence indicates that each administration method may present its own pros and cons (11). Since the current evidence regarding the effects of the two administration methods was limited, clinicians may tend to select the method with a lower risk of adverse events. Therefore, in this comprehensive and up to date meta-analysis, we revealed that there was no statistically significant difference in the incidence of other feeding intolerance symptoms, such as vomiting, gastric retention, and aspiration pneumonia. Furthermore, despite intermittent feeding allowing for more time for patients to engage in rehabilitation training, which can be beneficial for recovery, the ICU mortality rate was higher in the intermittent feeding group. Even if the difference did not reach statistical significance, the result is of notable interest. A probable reason is the higher incidence of diarrhea and distension in patients receiving intermittent feeding may result in more severe complications. This result suggested that continuous feeding might be a preferable enteral nutrition administration strategy for critically ill adults to reduce feeding intolerance incidence, which is consistent with the recommendation of ASPEN guidelines (34). However, since both feeding methods may give rise to some form of feeding intolerances and gastroenterological complications, current practice should balance these potential adverse events for individualized patient care to mitigate potential adverse events (35). The latest consensus (36, 37) advocated shifting from the ‘one size fits all’ feeding approach to one where individualization may be the key factor in driving positive outcomes for critically ill patients. When determining individualized nutrition interventions for critically ill patients, the dietitian and clinicians should consider the patient population, the individual patient’s nutritional status and the expected outcome and formulate an appropriate feeding regimen based on this.

Some of the findings of our meta-analysis are in contrast to previous studies. A recent meta-analysis (35) showing no difference in mortality, diarrhea, increased gastric residuals, pneumonia, and bacterial colonization between intermittent and continuous enteral feeding methods. Ma et al. (14) reported a meta-analysis of 14 RCTs that found intermittent feeding provided significantly more calories, increased the risk of high gastric residual volume and aspiration, reduced the incidence of constipation as compared to continuous feeding. Nevertheless, the majority of studies included by Ma et al. (14) were predominantly local data where practices may differ from worldwide feeding practices, the results may not be fully appropriate and executable internationally. Compared with previous meta-analyses, our study performed a comprehensive and up to date search of the worldwide literature, included more recent RCTs (15, 27) that met the inclusion criteria. The results of our research are approximately consistent with the previous studies, but we found that the intermittent enteral feeding was associated with higher incidence of diarrhea and distension. The outcome measure for nutritional intake in our meta-analysis was the proportion of achievement the nutritional goal, which is a more meaningful measure of nutritional adequacy, as opposed to total calories.

Notably, the current study found a higher incidence of constipation in the continuous feeding group compared to the intermittent feeding group, which was consistent with the conclusion of previous studies (14, 35). This may be attributed to colonic sensorimotor disturbance, which is a well-established cause of constipation (38). Gut motility is partly dependent on stimulation from intraluminal contents, which is more effectively achieved through intermittent feeding that delivers a larger amount of intraluminal contents within a certain period, and can thereby stimulate intestinal smooth muscle contractions (39). Conversely, continuous feeding is delivered at a slower rate and with smaller volumes, which may not effectively stimulate postprandial patterns of gastrointestinal motility and lead to reduced antral hypomotility (8). Furthermore, prolonged bed rest is a proved risk factor for constipation (40), and continuous feeding patients are often restricted to bed due to the non-interruptible nature of enteral nutrition, which may also contribute to the higher incidence of constipation in this group (41).

In practice, various factors may influence the options of feeding modalities. Previous meta-analysis by Ma et al. (14) indicated that intermittent administration has been linked to a potentially higher daily caloric intake (MD 184.81, 95% CI 56.61 to 313.01), although our own study did not detect difference in the achievement of nutritional goal between intermittent and continuous feeding groups. Interruptions in enteral nutrition delivery due to patient care or diagnostic testing may contribute to this difference. Furthermore, intermittent administration may allow for faster achievement of nutritional goals, particularly in cases where continuous administration is gradually initiated. The impact of these factors on patient outcomes remains uncertain. Moreover, intermittent administration may have the added benefit of minimizing patient immobility associated with additional tubing, particularly in a hospital setting.

### Limitations

However, our meta-analysis has several limitations. First of all, due to the apparent distinction between the two feeding strategies, blinding of participants and investigators was difficult to implement. Our meta-analysis included RCTs with moderate to high levels of bias and small sample sizes (number of participants <100 per arm), thereby limiting the scope of definitive conclusions that can be drawn (31). Hence, caution must be exercised while interpreting the findings of such studies.

Second, included studies varied in the definitions for assessing the outcomes of interest (e.g., distension, gastric retention, and aspiration pneumonia), which could cause indirectness of evidence. Such heterogeneity in outcome definitions warrants careful consideration while interpreting and synthesizing the results from these studies.

Moreover, several studies did not provide sufficient details regarding the determination of the nutritional requirements of the patients, the formulas employed for the calculation of these requirements, and other factors that may influence these calculations, including the potential inclusion of propofol. The absence of such crucial information may undermine the ability to fully appraise the study findings.

## Conclusion

In this updated meta-analysis of 13 RCTs, we found that intermittent enteral feeding in critically ill patients was associated with high occurrence of feeding intolerance, including diarrhea and distension. However, there is a higher risk of constipation associated with continuous enteral feeding. Based on available evidence, continuous enteral feeding may be more appropriate for patients at higher risk of feeding intolerance. Moreover, we advocate for additional high-quality RCTs with longer durations and more diverse clinical outcomes to more effectively validate the effects of both continuous and intermittent feeding methods on enteral nutrition.

## Data availability statement

The original contributions presented in the study are included in the article/Supplementary material, further inquiries can be directed to the corresponding author.

## Author contributions

JQ and LH conceived the idea, performed the analysis, and drafted the initial draft writing of this paper. XX, CX, and XD contributed to the collection and interpretation of data. KZ helped to frame the idea of the study and provided technical support. LH contributed to the revision of this paper, and the final approval of the version to be published. All authors contributed to the article and approved the submitted version.

## Conflict of interest

The authors declare that the research was conducted in the absence of any commercial or financial relationships that could be construed as a potential conflict of interest.

## Publisher’s note

All claims expressed in this article are solely those of the authors and do not necessarily represent those of their affiliated organizations, or those of the publisher, the editors and the reviewers. Any product that may be evaluated in this article, or claim that may be made by its manufacturer, is not guaranteed or endorsed by the publisher.

## Footnotes

^1^ https://osf.io/3au84
