# Supplementary material for: The effect of intermittent versus continuous enteral feeding for critically ill patients: a meta-analysis of randomized controlled trials
Source: Front Nutr. 2023 Aug 21;10:1214774. doi: 10.3389/fnut.2023.1214774 (PMC10475573; doi:10.3389/fnut.2023.1214774)
Supplement: Supplementary file 3 [file Data_Sheet_3.docx]

**Supplementary Material 3:** Publication bias assessment by funnel plot and Egger’s test, sensitivity analyses.


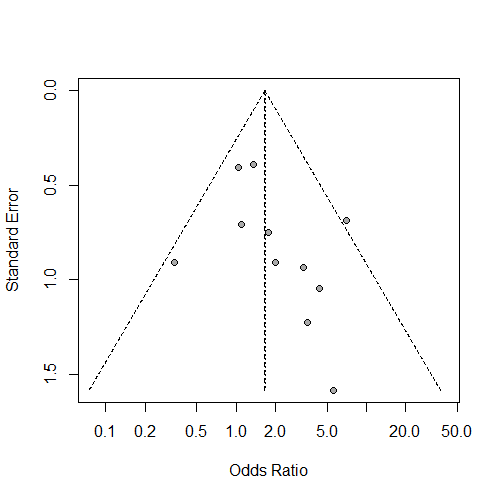


Figure 1: Funnel plot for diarrhea, Egger’s test P=0.1930


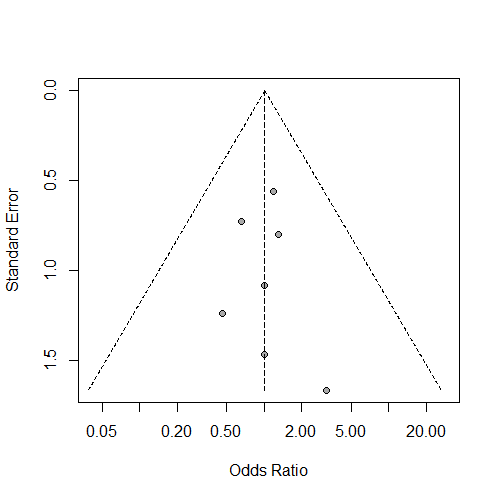


Figure 2: Funnel plot for vomiting, Egger’s test P=0.9048


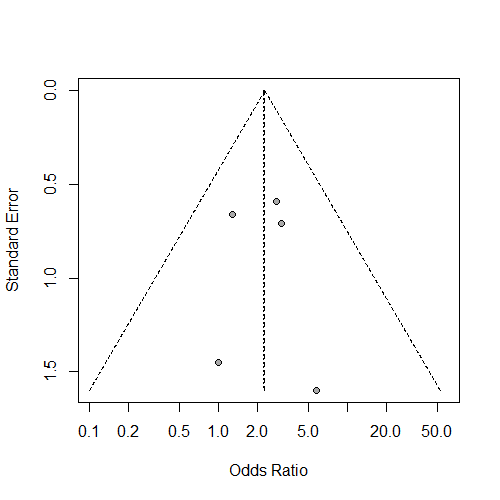


Figure 3: Funnel plot for distension, Egger’s test P= 0.9565


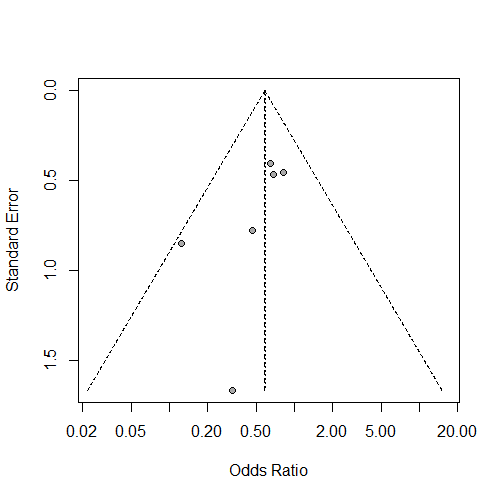


Figure 4: Funnel plot for constipation, Egger’s test P=0.1468


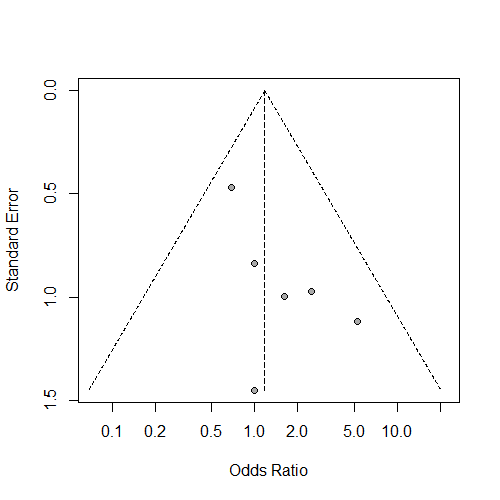


Figure 5: Funnel plot for gastric retention, Egger’s test P=0.0926


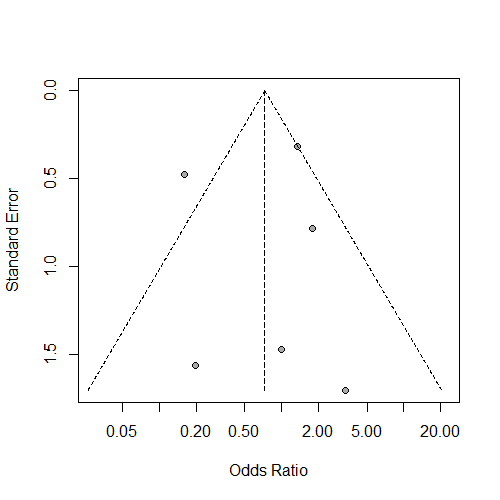


Figure 6: Funnel plot for aspiration pneumonia, Egger’s test P=0.9230


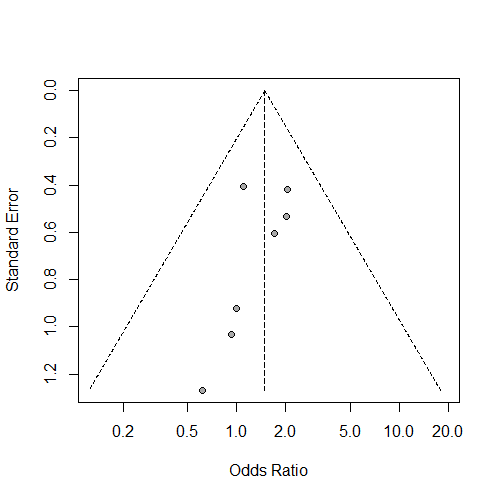


Figure 7: Funnel plot for mortality, Egger’s test P=0.2537


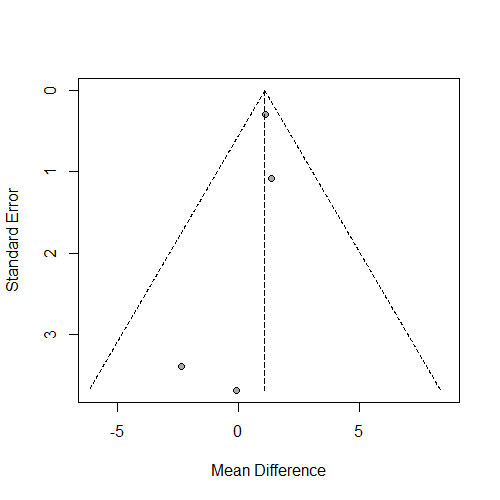


Figure 8: Funnel plot for length of ICU stay, Egger’s test P=0.3351


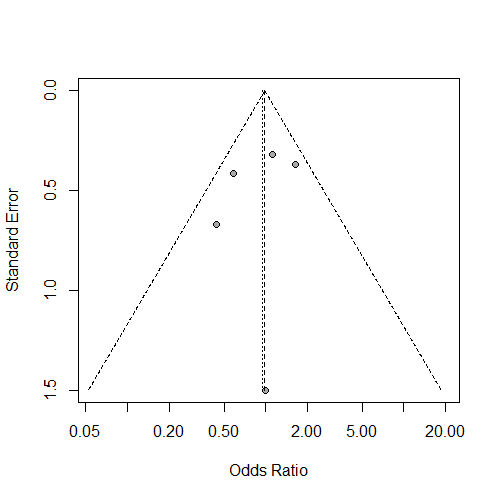


Figure 9: Funnel plot for achievement of nutritional goal, Egger’s test P=0.5425


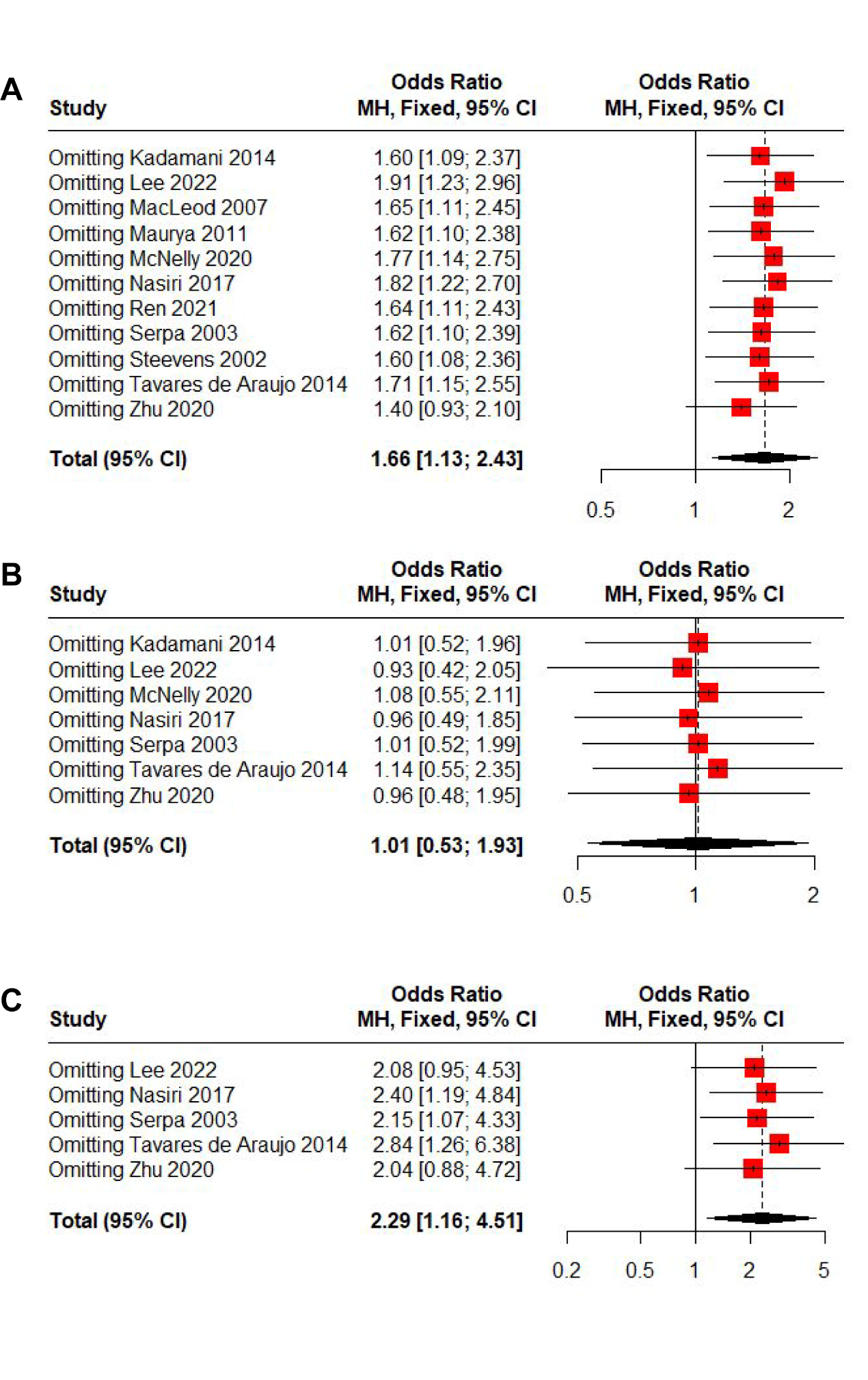


Figure 10: Sensitivity analysis for (A) diarrhea, (B) vomiting, (C) distension


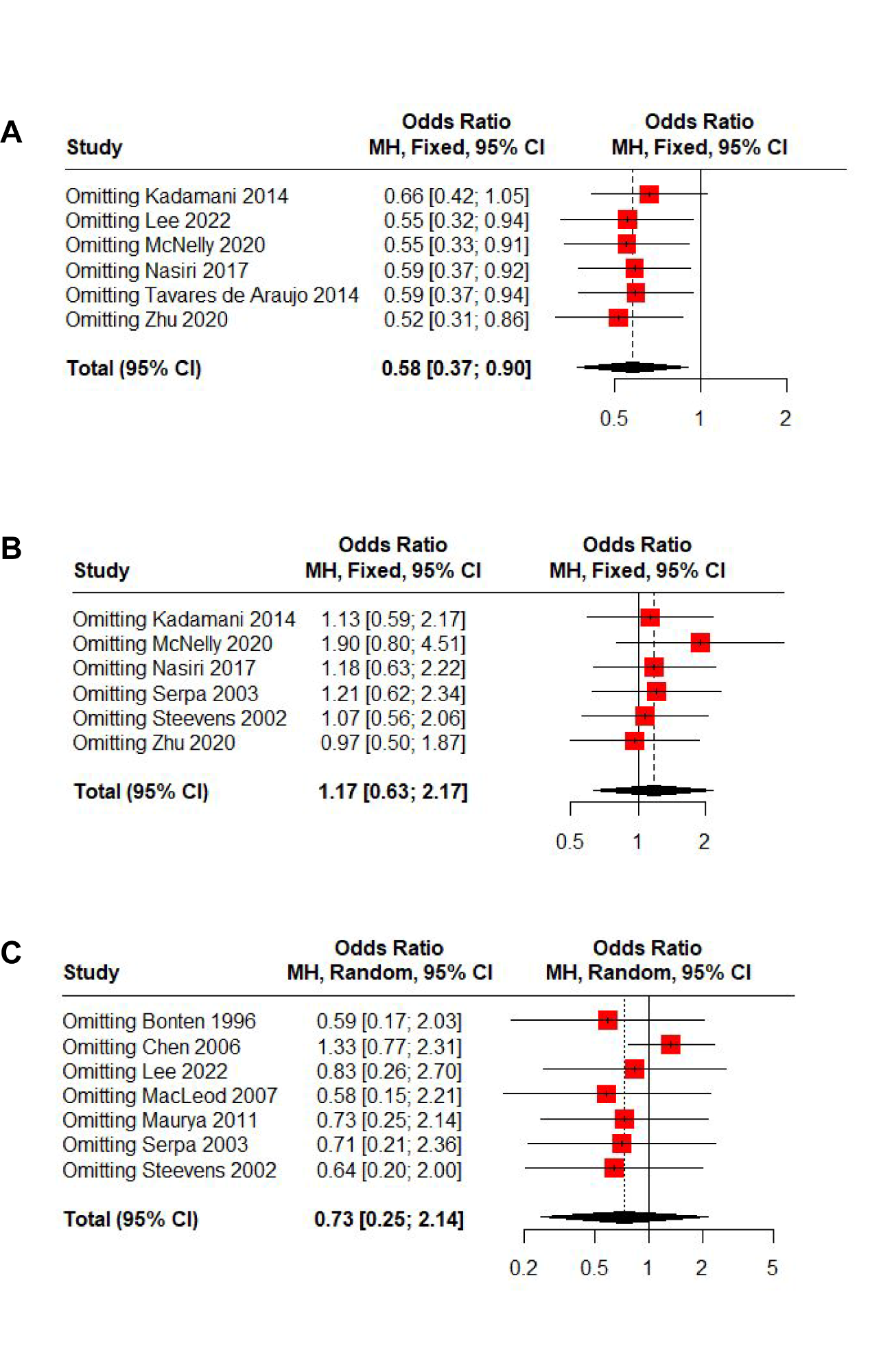


Figure 11: Sensitivity analysis for (A) constipation, (B) gastric retention, (C) aspiration pneumonia


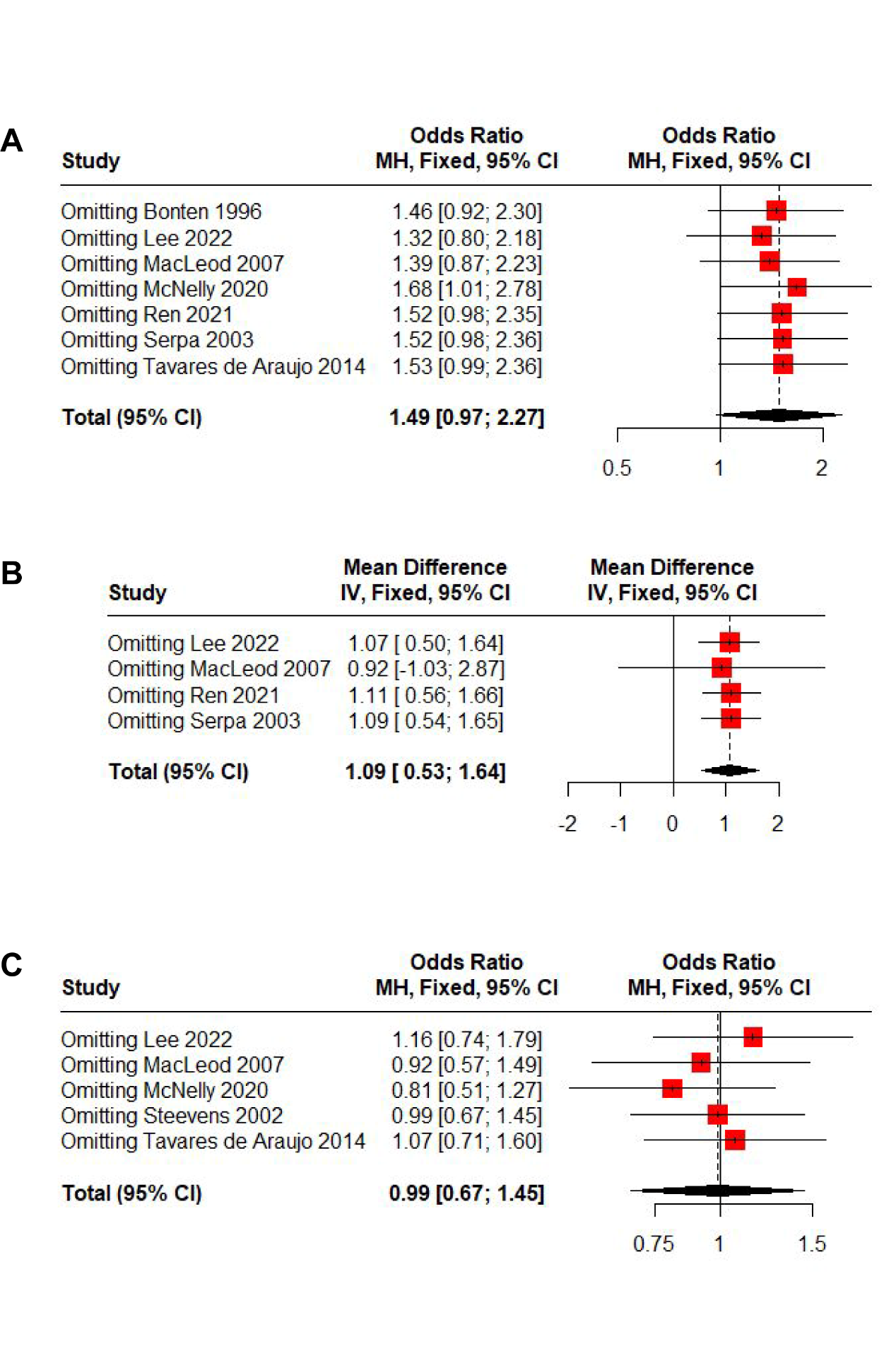


Figure 12: Sensitivity analysis for (A) mortality in ICU, (B) length of stay in ICU, (C) achievement of nutritional goal
