# Supplementary material for: The effect of intermittent versus continuous enteral feeding for critically ill patients: a meta-analysis of randomized controlled trials
Source: Front Nutr. 2023 Aug 21;10:1214774. doi: 10.3389/fnut.2023.1214774 (PMC10475573; doi:10.3389/fnut.2023.1214774)
Supplement: Supplementary file 4 [file Data_Sheet_4.docx]

**Supplementary Material 4:** Definitions of outcomes in each included trial

| Study | Outcomes and definition |
| --- | --- |
| Lee et al. 2022 | Feeding intolerances: diarrhea, vomiting, constipation, distension, aspiration;  Achievement of nutritional goal: achievement ≥80% of the target nutrition requirement;  ICU mortality: mortality in ICU;  Length of ICU stay: number of days stay in ICU |
| Ren et al. 2021 | Feeding intolerances: diarrhea (three or more loose stools or continued to have loose stools within 24 hours);  ICU mortality: mortality in ICU;  Length of ICU stay: number of days stay in ICU |
| Zhu et al. 2020 | Feeding intolerances: diarrhea (liquid stool over three times a day), vomiting, distension (abdominal circumference increased 3 cm or more), constipation (patients did not defecate for 3 days), retention (200 mL of fluid or more could be suctioned) |
| McNelly et al. 2020 | Feeding intolerances: diarrhea (Bristol Stool Chart score ≥5), vomiting, constipation (patients did not defecate for 3 days), retention (gastric residual volume ≥300 mL), aspiration  Achievement of nutritional goal: achievement ≥80% of the target nutrition requirement;  ICU mortality: mortality in ICU |
| Nasiri et al. 2017 | Feeding intolerances: diarrhea, vomiting, constipation, distension, retention |
| Kadamani et al. 2014 | Feeding intolerances: diarrhea (patient passed 3 or more times of loose stool per day); vomiting, constipation (patient had absent bowel movement for three consecutive days or more), retention (aspirated gastric residual volume ≥200 ml) |
| Tavares de Araujo et al. 2014 | Feeding intolerances: diarrhea, vomiting, distension, constipation;  Achievement of nutritional goal: achievement the nutritional needs;  ICU mortality: mortality in ICU |
| Maurya et al. 2011 | Feeding intolerances: diarrhea, aspiration |
| MacLeod et al. 2007 | Feeding intolerances: diarrhea, aspiration;  Achievement of nutritional goal: reach the nutrition goal;  ICU mortality: mortality in ICU;  Length of ICU stay: number of days stay in ICU |
| Chen et al. 2006 | Feeding intolerances: aspiration (glucose in sputum, chest radiography, wheezing, fever) |
| Serpa et al. 2003 | Feeding intolerances: diarrhea, vomiting, distension (abdominal circumference increased 3 cm or more), retention (150 mL of fluid or more could be recovered immediately before each 3-hour period), aspiration (inspecting tracheal secretions for any blue colorations during tracheal suctioning);  ICU mortality: mortality in ICU;  Length of ICU stay: number of days stay in ICU |
| Steevens et al. 2002 | Feeding intolerances: diarrhea (stool volume ≥250 mL, or ≥3 loose stools per day), retention (gastric residual volume ≥250 mL), aspiration (inspecting tracheal secretions for any blue colorations during tracheal suctioning);  Achievement of nutritional goal: achieve the nutrient goal |
| Bonten et al. 1996 | Feeding intolerances: aspiration;  ICU mortality: mortality in ICU |
